# HABscope: A tool for use by citizen scientists to facilitate early warning of respiratory irritation caused by toxic blooms of *Karenia brevis*

**DOI:** 10.1371/journal.pone.0218489

**Published:** 2019-06-20

**Authors:** D. Ransom Hardison, William C. Holland, Robert D. Currier, Barbara Kirkpatrick, Richard Stumpf, Tracy Fanara, Devin Burris, Andrew Reich, Gary J. Kirkpatrick, R. Wayne Litaker

**Affiliations:** 1 National Oceanic and Atmospheric Administration, National Ocean Service, Center for Coastal Fisheries and Habitat Research, Beaufort, North Carolina, United States of America; 2 Gulf of Mexico Coastal Ocean Observing System, Department of Oceanography, Texas A & M University, College Station, Texas, United States of America; 3 National Oceanic and Atmospheric Administration, Center for Coastal Management and Assessment, Silver Spring, Maryland, United States of America; 4 Mote Marine Laboratory and Aquarium, Sarasota, Florida, United States of America; 5 Florida Department of Health, Public Health Toxicology Section, Tallahassee, Florida, United States of America; University of Connecticut, UNITED STATES

## Abstract

Blooms of the toxic microalga *Karenia brevis* occur seasonally in Florida, Texas and other portions of the Gulf of Mexico. Brevetoxins produced during *Karenia* blooms can cause neurotoxic shellfish poisoning in humans, massive fish kills, and the death of marine mammals and birds. Brevetoxin-containing aerosols are an additional problem, having a severe impact on beachgoers, triggering coughing, eye and throat irritation in healthy individuals, and more serious respiratory distress in those with asthma or other breathing disorders. The blooms and associated aerosol impacts are patchy in nature, often affecting one beach but having no impact on an adjacent beach. To provide timely information to visitors about which beaches are low-risk, we developed *HABscope*; a low cost (~$400) microscope system that can be used in the field by citizen scientists with cell phones to enumerate *K*. *brevis* cell concentrations in the water along each beach. The HABscope system operates by capturing short videos of collected water samples and uploading them to a central server for rapid enumeration of *K*. *brevis* cells using calibrated recognition software. The HABscope has a detection threshold of about 100,000 cells, which is the point when respiratory risk becomes evident. Higher concentrations are reliably estimated up to 10 million cells L^-1^. When deployed by volunteer citizen scientists, the HABscope consistently distinguished low, medium, and high concentrations of cells in the water. The volunteers were able to collect data on most days during a severe bloom. This indicates that the HABscope can provide an effective capability to significantly increase the sampling coverage during *Karenia brevis* blooms.

## Introduction

*Karenia brevis* blooms occur annually along the West Coast of Florida, USA and can occur along other U.S coastal states from the Texas coast to North Carolina. These blooms consistently produce a suite of potent neurotoxins (brevetoxins) that bind to voltage-gated sodium channels and cause disruption of cellular function, particularly in muscle cells [1]. Brevetoxins become aerosolized following the breakage of *Karenia brevis* cells by surf or wind shear and are transported onshore by prevailing winds [2]. Once inhaled, these aerosolized toxins trigger bronchoconstriction and smooth respiratory muscle responses that result in asthma attacks in susceptible individuals and those with chronic obstructive pulmonary disease [3–8]. This is important because asthma afflicts about 9% of the U.S. population [9]. Even minute levels of brevetoxin (0.5 ng m^-3^) cause a negative health response in people with asthma [10]. When aerosolized brevetoxins are sufficiently high, even less sensitive individuals experience symptoms that include a non-productive cough, nasal congestion, and a strong burning sensation in the eyes and nose. Hospital records from Florida indicate emergency room visits for respiratory illness increase during these red tides, elevating medical costs and the burden to the local medical community. For Sarasota County, Florida alone, these additional emergency room visits cost between US $0.5 and $4 million, depending on the severity of the bloom [11, 12]. Other adverse effects such as lost tourism and restaurant business, as well as resources necessary for removal of dead fish from beaches, etc. can cost up to US $25 million in additional losses. Loss of tourism and business revenue from tourists relocating and seeking alternative vacation locations during *Karenia brevis* blooms can be up to 36% of the total expected revenues [13].

The amount of aerosolized brevetoxins in the air is highly variable from day to day with typical levels ranging from 15 to 90 ng m^-3^ [14]. This variability is due to the complex interaction of prevailing winds and hydrodynamic conditions that bring *K*. *brevis* blooms onshore in localized patches. Consequently, beaches a few kilometers apart may experience very different toxic aerosol levels. Some beaches will be unaffected, while beachgoers only a few km away can experience severe respiratory irritation. The prevailing wind direction is also critical. A beach with an intense bloom can be pleasant to enjoy with a strong offshore wind in the correct orientation. To complicate matters further, during strong onshore winds, exposure can occur not only at the beach, but also one km or more inland [15]. Pre-medication for asthma and air conditioning (air filtration) are effective means of treating adverse symptoms [14], provided timely information about bloom severity is available to allow planning in advance. If the public is to be protected adequately against the risk of brevetoxin exposure, it is critical that daily, beach-specific bloom forecasts are easily accessible.

Current monitoring for *K*. *brevis* blooms involves two primary components. First, satellite imagery is routinely processed to locate potential blooms. These processed images are made available to managers and state health officials in the Gulf of Mexico through the National Oceanic and Atmospheric Administration (NOAA) Harmful Algal Bloom Operational Forecast System (HAB-OFS, https://tidesandcurrents.noaa.gov/hab/overview.html). Second, in the case of Florida where blooms tend to occur most frequently, water samples are collected weekly along the shore and from offshore transects by the state Fish and Wildlife Research Institute once a bloom is identified. Samples must be delivered to a laboratory for cell enumeration via microscopy. Including the time required for sample settling, microscopic enumeration takes about one hour per sample. Typically, samples are processed within 1–2 days and can take longer for more samples from remote areas. The resulting cell counts are then used by HAB-OFS to provide broad, county-wide forecasts of brevetoxin exposure risks. This approach is overly conservative and the data for a particular county can be up to a week old by the time it is available to the public. Stumpf et al. [16] found that while county-wide forecasts of respiratory risk are correct >70% of the time, they were correct only 20% of the time when applied to individual beaches.

To address the need for location-specific conditions, a Beach Conditions Reporting System (BCRS) was initiated in 2006 [16–19]. The BCRS provided smartphones to (professional) lifeguards and park rangers with an app designed for reporting beach conditions. Twice each day (1000 h and 1500 h local time), lifeguards and park rangers report occurrence of coughing (and other conditions like presence of dead fish). While lacking the quantification and precision of microscopy, the reports provide beachgoers with useful information for adequate planning (i.e. severity of aerosolized toxins, potential risks to asthmatics, presence of dead fish, etc.). The BCRS has been continued by Mote Marine Laboratory, Florida with lifeguards and park rangers in several Florida counties (https://visitbeaches.org). The BCRS data collection is automated, with timely sharing of data with agencies, including NOAA.

Though the BCRS provides more timely information about beach conditions than the weekly sampling, it does not provide key information needed for consistent forecasts. It provides no information on *K*. *brevis* cell presence, only a general statement about water color, which may become darker during a bloom. Because many other factors can change water color, this information has limited value. If winds are blowing offshore in the morning, the public will not know whether the beach will be usable in the afternoon, when there may be a sea breeze onshore. The potential for expanding the system is also limited as lifeguards are posted only at designated county beaches. For example, over the 50 km of beaches in Sarasota County there are only nine BCRS sites. Other Florida counties have even fewer sites.

Ideally, the Gulf of Mexico region needs a monitoring system designed to collect daily samples of cell concentration from every beach. “Every Beach, Every Day” is the motto for this monitoring project, for which the objective is to provide daily cell abundance data from every beach in Florida that is affected by *Karenia brevis* blooms. These abundance data would allow production of daily beach-specific reports and forecasts that the public could use to select a beach and time of day with the lowest risk of respiratory impacts. To do this sampling with traditional manual cell counting would be prohibitively expensive because of the time and resources required. Our approach to overcome this problem was to design a capability that brings the microscope to the field with volunteer citizen scientists, and use feature recognition software to retrieve the data.

Citizen science empowers ordinary people to be stewards of their own environment and leaders of conservationism in their community. As a specific example involving *Karenia brevis*, the *Texas Red Tide Rangers* are a network of volunteers that are part of the Texas Master Naturalist program, and are trained in traditional microscopy to identify *Karenia brevis* (https://www.utrgv.edu/csl/public-service/red-tide/index.htm). The Rangers can then send samples to state labs for confirmation and enumeration. In Florida, Mote Marine Laboratory has a successful 20-year history with their volunteer network of citizen scientists who actively monitor assigned beaches for such concerns as sea turtle nests. This long experience and excellent local reputation makes volunteers abundantly available in the Mote region and facilitates establishment of a training program for *K*. *brevis* bloom monitoring. The impact of this approach is a finer scale of sampling resolution than can be achieved by traditional monitoring by scientists. Another successful citizen science program is *eBird*, a volunteer network maintained by the Cornell Lab of Ornithology. eBird volunteers share their birding experiences though the organization’s website (https://ebird.org/home), allowing scientists with the ability to track migration, nesting and mating patterns of different bird species [20]. A similar example of citizen science was used in the risk assessment of wind farms to bats in southern Scotland [21]. In this application, citizens were outfitted with an acoustic device to determine location and species of bats in the proposed wind farm areas. Citizen science increases the geographic scope and frequency of data, and provides increased participant numbers and geographical diversity as well as a simple, reliable data collection device [22].

For such a citizen science effort to be successful in providing routine data on *K*. *brevis* from individual beaches, volunteers must be provided with a fast and easy way of estimating *K*. *brevis* cell numbers. Once known, these cell concentrations can be combined with information on wind speed and direction to estimate brevetoxin respiratory exposure risk. This article details the development of the “HABscope” system to provide citizen scientists the ability to rapidly determine *K*. *brevis* cell counts. Also discussed is an evaluation of how the system performs in the hands of citizen scientists who had received several hours of training.

## Materials and methods

The sampling device for determining *K*. *brevis* cell counts by citizen scientists is termed the HABscope. The design consists of a low cost, classroom laboratory grade, upright microscope fitted with a wireless iPOD touch, a brand of iOS-based mobile device (Apple, Inc., https://www.apple.com/ipod-touch) with a touchscreen-controlled user interface. The field HABscope is portable and includes a battery pack for the light source. Citizen scientists collect a water sample and use the iPOD adapted microscope to take a short video of the sample, which is then uploaded via mobile hotspot to the Gulf of Mexico Coastal Ocean Observing System (GCOOS) website (S1 Video). The video is analyzed using a neural network program trained to recognize *K*. *brevis* cells. Cell recognition is based on the size, unique shape and characteristic corkscrew swimming pattern of *K*. *brevis*, which helps distinguish it from co-occurring species typically found during *K*. *brevis* blooms. The HABscope system, including the iPOD and a fully capable binocular microscope, can be assembled for a total retail cost of only $400 USD. Other mobile phone-based microscope systems are available commercially or through custom order [12, 13], but cost considerably more (~$1,000 - $3,000 USD). This individual cost becomes important when considering how to expand the citizen science network to provide maximal coverage of individual beaches.

**Fig 1:**
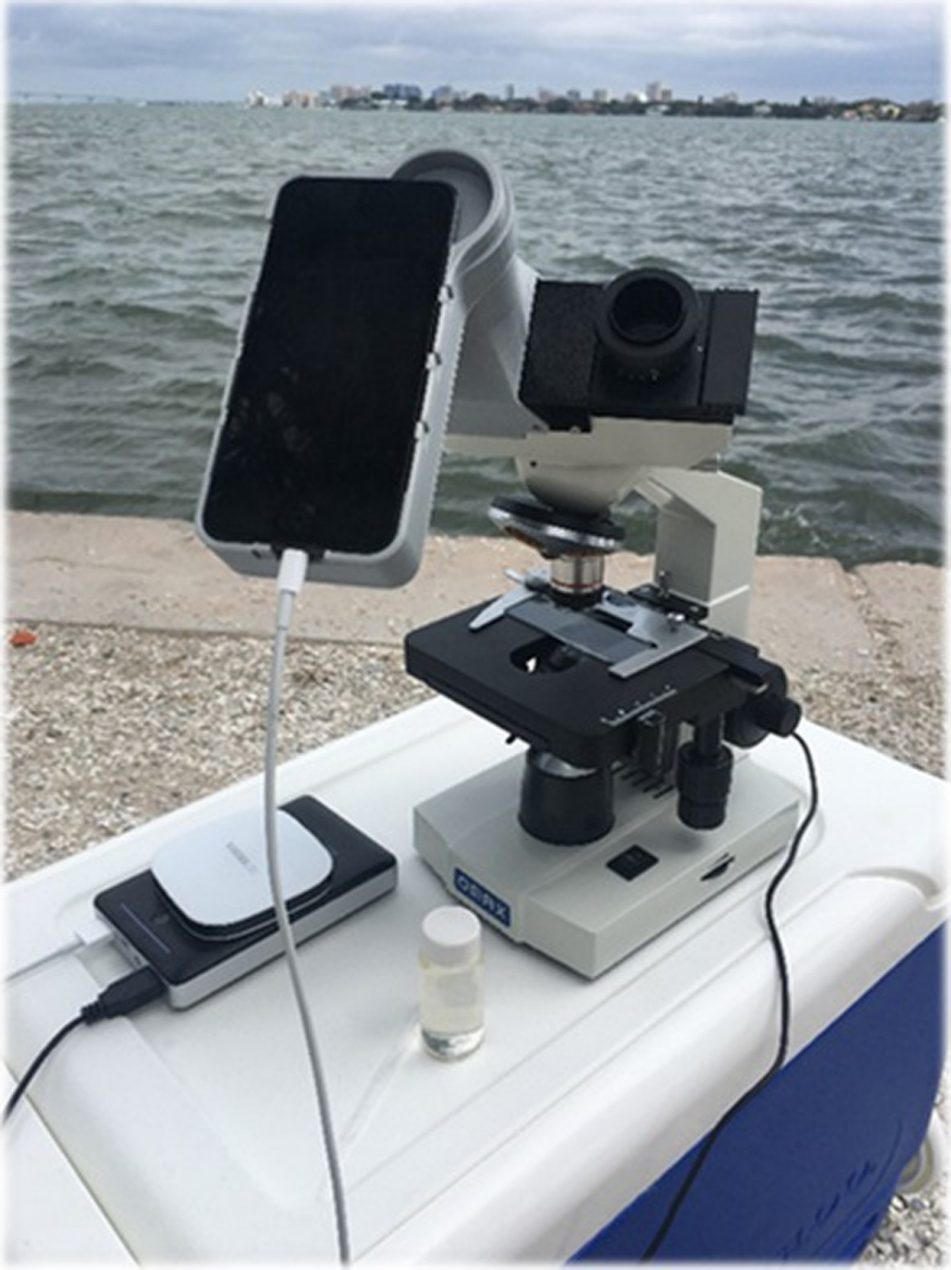
The HABscope setup. A photograph exhibiting the HABscope components, which are listed in Table 1 along with a list of suppliers and the 20 mL sampling vial.

The specific components of the HABscope system are as follows and were built with commercially available products except for an adaptor to mount the Apple iPOD touch (model MKHV2LL/A) to the eyepiece of the microscope (Fig 1). The core of the system used an OMAX microscope (OMAX microscope Corporation, Gyeonggi-do, Korea), with 4X, 40X and 200X objectives. Adaptors to hold the iPOD in alignment with the right side eyepiece of the OMAX microscope were printed using an Ultimaker 2+ three dimensional printer (Ultimaker B.V, Geldermalsen, The Netherlands) using 2.85mm polylactic acid (PLA; Ultimaker) (S1 and S2 File). Visual images and videos were taken with a 6^th^ generation Apple iPOD touch (Independence, Ohio, USA). Since the HABscope is field deployable, power was supplied for the microscope’s light source using an external 16750-mAh power source (RAVPower Model RP-PB10, https://www.ravpower.com) (Table 1). All connections were made using USB 2.0 ports and a 4 port USB hub (Sabrent, https://www.sabrent.com) (Table 1) to distribute power to the microscope. Each citizen scientist used their own personal hotspot option on their cellular phone to enable videos to be sent wirelessly to the GCOOS website for analysis.

**Table 1:** Parts and suppliers list for HABscope components.

Part	Manufacturer	Location	Model / Part number
OMAX microscope with 4X -200X objectives	OMAX	Gyeonggi-do, Korea	CS-M82ES-SC100-LP50
6^th^ generation Apple iPOD touch	Apple	Cupertino, California, USA	MKHV2LL/A
16750-mAh external power source	Ravpower, Inc.	San Jose, California, USA	Model RP-PB10
4 port USB hub	Sabrent	Los Angeles, California, USA	HB-UMLS
3 each USB 2.0 connection wires	Warehouse Deals, LLC	Juneau, Alaska, USA	53832-A-5337
Omano 10 microscope well slides, 1.5 cm depression	Microscope.com	Roanoke, Virginia, USA	OMWS-10PC
Cell counter vials	Beckman Coulter	Brea, California USA	14310–684
Lens paper wipes	Kimberley-/Clark	Irving, Texas, USA	34155
Pipettes- disposable, 1 mL	VWR	Georgia-Radnor, Pennsylvania, USA	16001–778

### Training the HABscope system

The HABscope system quantifies *K*. *brevis* cell abundance using a convolutional neural network (CNN) model, a deep learning computer program used for analyzing and identifying visual imagery. Cell identification criteria were based solely on detecting the swimming motion of the *Karenia* cells, with stationary objects ignored by the software. The CNN model used in HABscope is comprised of an input layer, three convolutional layers, a fully connected layer and an output layer. The activation function for all layers except the output layer is a ReLU (rectified linear unit). A sigmoid activation function is used for the output layer due to this being a binary cross-entropy model. The output layer is configured as Dense(1) as is appropriate for a binary classification problem. TensorFlow, an open-source library to develop and train machine learning models, was used to construct the model (http://www.tensorflow.org). Teaching the image recognition software to identify *K*. *brevis* was an iterative process. Initially, laboratory cultures of *K*. *brevis* were used to train the feature recognition software.

The next phase involved training the model to better distinguish *K*. *brevis* from other similar sized, free-swimming dinoflagellates. Approximately 5,993 images of *K*. *brevis*, *Amphidinium* sp. and *Alexandrium catenella* were acquired to allow the CNN to train in order to recognize the morphological differences between *K*. *brevis* and the other species. Because the HABscope CNN is configured as a binary cross-entropy model using rmsprop as the optimizer, the trained network classified cells into one of two classes: ‘*K*. *brevis’* or ‘*not K*. *brevis’*. The images classified as *not K*. *brevis* are indicated by a red target marker. A convolutional neural network does not have the ability to ‘know if it is right or wrong’, it simply reports the likelihood that the image in question has a higher or lower probability of matching one of two categories upon which it has been trained. The Keras Python library (https://keras.io/) then encodes the 0.0 to 1.0 answer returned by the CNN into a 1 or 0, using 0.5 as the threshold. If the CNN reports a 0.6 Keras returns a 1, if the CNN reports a 0.4 Keras, returns a 0. The ability of the discriminatory function to correctly classify *K*. *brevis*, and to classify *Amphidinium* sp. and *Alexandrium catenella* as *not K*. *brevis* was confirmed using mixed cultures of the three species where the relative proportion of *K*. *brevis* to the other species was known. Next *K*. *brevis* cells were spiked into the natural water samples in order to test the ability of the system to discriminate *K*. *brevis* cells from other material. It became apparent that particles of detritus moved around by microscope vibrations were sometimes misclassified as *K*. *brevis*.

To address this issue, the CNN was trained using additional images of detritus and *K*. *brevis* from natural samples as described above. The training library was updated over a five month period as new and/or better images were acquired. Periodically, these newly acquired images were used to retrain the CNN, with the updated model being stored thereafter. As of 2019-04-22, the training library includes 15,666 images of *K*. *brevis* and 5,993 images of detritus. The validation library has 3,637 images of *K*. *brevis* and 2,687 images of detritus and non *K*. *brevis* cells. In all, a total of 58,819 images were collected for the training purposes. A final production model was created with a training run of 50 iterations. Both the training and model validation was accomplished using a computer equipped with an MSI GeForce GTX 1080 Ti GPU, 32GB of RAM and an Intel Core i7-6850K processor (MSI Computer Corp., City of Industry, California, USA).

### Estimating cell concentration using the CNN model

An image analysis pipeline was developed to estimate cell concentrations. Short, 30 second videos were taken of laboratory cultures containing varying, but known, concentrations of *K*. *brevis* using the HABscope and iPod setup. In total, 35 videos were analyzed. Cell concentrations in each *K*. *brevis* culture was determined using a Beckman Coulter Multisizer 3 particle counter (Beckman Coulter, Inc., Brea, California, USA). A 280 μm aperture tube and 1 mL sample volume were utilized for particle counts. Concentrations ranged from 50,000 cells L^-1^ to 30,000,000 cells L^-1^. The resulting videos were then uploaded to the network and subsequently run through the OpenCV vision and machine learning software library (http://opencv.org). Each video was analyzed frame by frame for areas in motion. When a region of interest was identified, it was compared to a list of known morphological characteristics of the model taxa. If the region of interest (ROI) was determined to match the model taxa, the ROI was clipped from the video frame and fed to the deep learning model. The deep learning model was built using Python language (https://www.python.org), TensorFlow open source numerical computation library, and the Keras high-level neural network application programming interface (API). The output was a ‘self-learned’ scale of visible cells ranging from zero to two hundred cells. By comparing the ‘self-learned’ cell counts for each culture with the corresponding known cell counts, it was possible to develop a scale for translating the processed HABscope videos into actual cell counts over the range of zero to thirty million cells L^-1^.

### Testing the system in the field

In 2017, the HABscope system was tested by having scientists sample an ongoing bloom at Mote Marine Lab’s New Pass (27°20’01.67" N, 82°34’44.60" W) and Bay (27°19’53.89" N, 82°34’39.67" W) docks. Special permission from Mote Marine Laboratory was granted to take water samples at these dock locations. Surface seawater samples were collected in 20 mL vials (USA Scientific, Inc., Ocala, Florida, USA). Immediately after the water samples were collected, the capped vials were gently mixed by slow inversion several times to ensure cells were in suspension and three drops were added to a 100 μL depression slide with a dropper pipette and examined with the microscope. Autoexposure of the iPOD was locked and the camera was adjusted to full zoom. Once cells were focused on the microscope at 40X, a 30 s video was taken and uploaded via hotspot to the GCOOS website. The video was automatically analyzed using the neural network software and a cell estimate was produced. Along with uploaded videos, the HABscope system also recorded latitude, longitude and local time from the iPOD’s GPS.

To determine the accuracy of the HABscope count, the remainder of each sample was preserved using several drops of neutral Lugol’s iodine solution [23, 24]. The preserved cells were transported to the laboratory and stored at 4°C until they were counted manually. For manual counting, each vial was mixed by inverting the vial several times and gently transferring the entire sample to a single well in a 6-well Falcon polystyrene tissue culture plate (Fisher Scientific, Pittsburgh, Pennsylvania, USA). Cells were allowed to settle for several hours, and then counted using an inverted microscope. The bottom of the plate was systematically scanned and the cells were tallied on a hand counter (Bates, MSC Industrial Supply Co., Melville, New York, cat. no. 63595839). If 400 cells were reached, the ratio of the surface area counted to total well surface area was used to estimate the total cell concentration. The manually counted samples were then compared to the counts for corresponding samples estimated using the HABscope.

### Volunteer training and sample collection

Mote Marine Laboratory and Aquarium (MMLA, Sarasota, Florida, USA, https://mote.org) has a long history of employing citizen volunteers at the aquarium, as docents in monitoring and rehabilitation programs for sea turtles and marine mammals, and as laboratory research assistants. This capability has made MMLA an ideal partner for the citizen science network needed for the current project. Citizen scientists were recruited through a variety of MMLA outreach events discussing the impacts of *K*. *brevis* blooms, the need for increased monitoring to improve HAB forecasts, and an overview of the HABscope. Volunteers were selected based on the proximity of their homes to affected beaches, year-round residence in the area, and a commitment to collect observations daily. Citizen scientists received intensive training at MMLA over a 2–3 day period, depending on their individual skill level. Background information on *K*. *brevis* blooms was reviewed and instruction on the use of the HABscope was provided by MMLA staff. A detailed user manual and step-by-step checklist was also made available. After the training, volunteers were provided with a HABscope and supplies (sample bottles, pipettes, microscope slides, etc.). Citizen scientists were asked to provide at least one daily video collected in the morning prior to peak beach visitation hours (10:00 am). Of the eight citizen scientists trained, two were asked to preserve water samples from Venice Beach (27°06’02.33" N, 82°27’38.41" W) and Nokomis Beach, Florida (27°07’25.76" N, 82°28’15.94" W) for verification with manual cell counts. No specific permit or permission was required for collection of water samples as these are public beaches. Subsequent to the training period, the number of times the volunteers provided HABscope samples was tracked to determine their ability to sample on a regular basis.

As soon as possible after sample collection, a subsample was mounted on the HABscope and a 30 second video was taken. Videos from the different sampling sites were uploaded to the GCOOS website, automatically analyzed using the neural network software, and an estimated cell concentration was produced within a five minute period. To assist with the citizen scientist training, and ensure quality control, all videos were screened visually prior to their use in estimating respiratory risk. This quality control step ensured videos were sufficient for obtaining accurate cell counts. Poor quality videos were rejected and the issues noted to assist with further citizen scientist training. Poor quality videos were most common when volunteers first began taking samples and typically involved field-of-view, focus, alignment or brightness issues. Following formal approval, the videos were immediately analyzed using the trained neural network to provide a cell estimate. From the point that the samples were collected, the time required for video upload, counting, review, and approval was typically between 30 minutes to one hour.

### Accuracy required of the HABscope system

An important question with respect to evaluating the HABscope performance is understanding how accurately the system must estimate *K*. *brevis* abundance to produce an acceptable respiratory risk assessment. Previous work demonstrated that cell concentrations below 100,000 cells L^-1^ pose low respiratory risks, whereas *K*. *brevis* concentrations between 100,000 cells L^-1^ to 1,000,000 cells L^-1^ and above 1,000,000 cells L^-1^ pose medium and high risks, respectively. Therefore, the HABscope system must be able to determine if *K*. *brevis* abundance at a given beach is < 100,000 cells L^-1^ (low-risk), between 100,000 cells L^-1^ and 1,000,000 cells L^-1^ (medium-risk), or > 1,000,000 cells L^-1^ (high-risk) in order to provide data sufficient for a respiratory forecast. Stumpf et al. [16] showed that for low, medium and high concentrations of *K*. *brevis* cells, there was an empirical relationship between wind speed and direction (relative to the prevailing shoreline orientation) and the degree of respiratory risk from aerosolized brevetoxins. As a result, high resolution wind speed and direction data, combined with estimated *K*. *brevis* abundance data, could provide forecasting capacity sufficient to gauge respiratory risk on individual beaches. Considering the availability of National Weather Service (NWS) high resolution (<2.5 km) wind speed and direction data, respiratory risk assessments based on local wind conditions could be provided at 3-hour intervals for up to 24 h after *K*. *brevis* cell counts are determined.

### Metrics

HABscope algorithm validation was assessed for bias and accuracy. Given the range of data, both were determined as percentages, accuracy was determined as mean absolute percentage error (MAPE) [25], and bias as mean percentage error. Over several orders of magnitude, MAPE is typically a better accuracy metric than mean absolute error, as error tends to be proportional to the magnitude. The current model for respiratory irritation is categorized as “low, medium, or high”, corresponding to the equivalent cell concentrations. The ability of the HABscope field retrievals was accordingly assessed as a classification confusion matrix (assesses performance of the classification algorithm), and percent accuracy for the three classes was determined. The Kappa statistic, which compares how well the classifier performed as compared to how it would have performed by chance, was calculated [26]. Kappa = 0 indicates the classification performs no better than random matches between the two classes, a Kappa = 1 indicates an exact match.

## Results

### Laboratory validation

**Fig 2:**
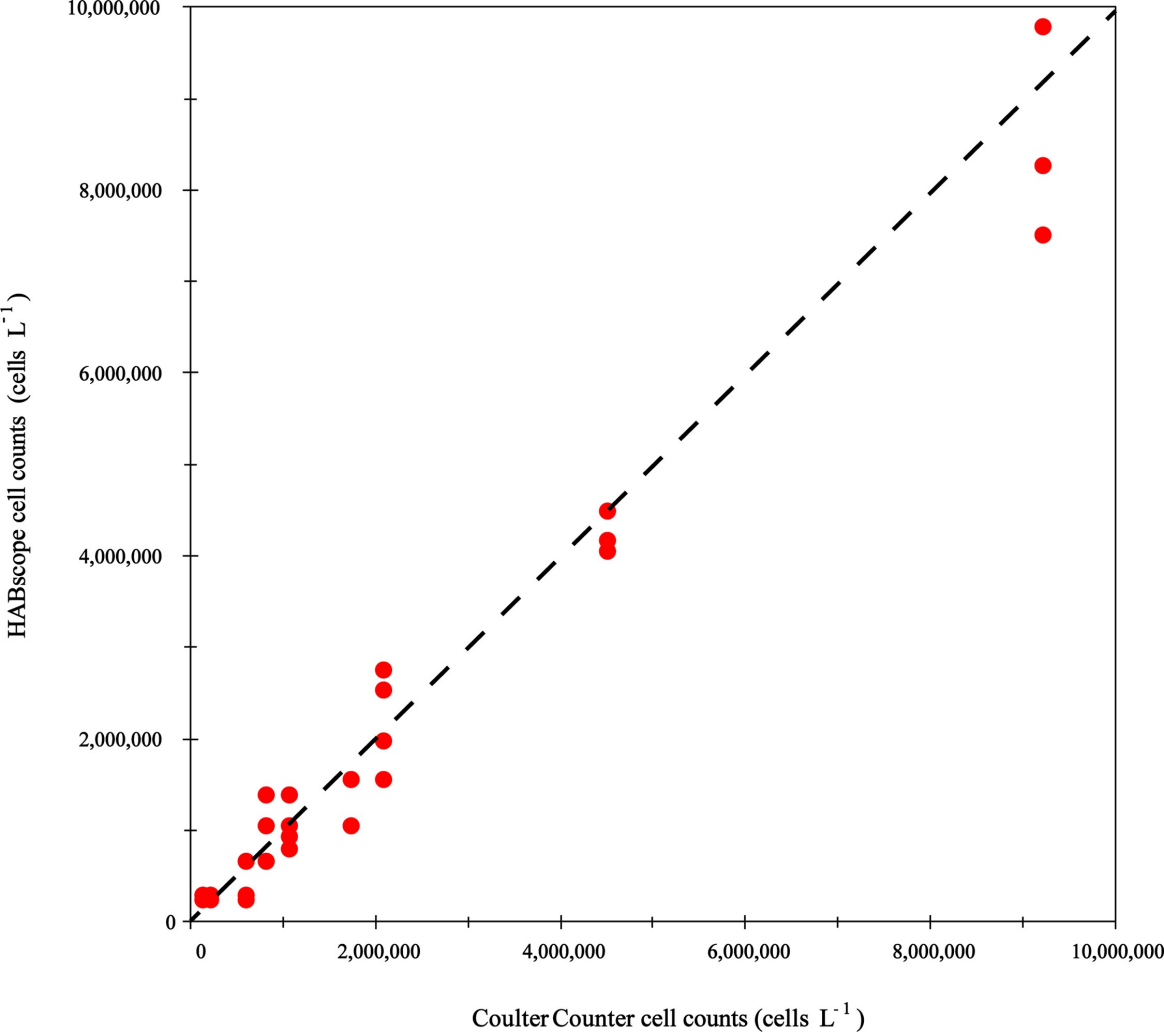
Laboratory validation of HABscope cell counts. A model validation of HABscope cell counts against varying concentrations of cultured *Karenia brevis* cells is shown. Cell concentrations determined with the HABscope (cells L^-1^) are plotted against those determined with a particle counter (cells L^-1^). The dashed line represents the 1:1 line.

A comparison between the cell abundances determined using the HABscope recognition software (cells L^-1^) with those determined with the particle counter (cells L^-1^) showed that the software was able to detect *K*. *brevis* cells with an accuracy (MAPE) of 31% over a range of 120,000 to 9,200,000 cells L^-1^ (Fig 2). This approximately spans the range of low, medium and high cell concentrations needed to assess respiratory risk. In comparison to cell concentrations determined with the Coulter Counter, the HABscope showed a linear response over this entire range but tended to over-estimate the lowest concentrations. There was no evident bias at other cell concentrations (Fig 2).

The limit of detection for the HABscope was determined using the number of cells visible in the field of view (FOV). At a magnification of 40X and a cell concentration of 50,000 *K*. *brevis* cells L^-1^, 1–2 cells are visible within the FOV. This means that the lower limit of detection for the HABscope is 50,000 *K*. *brevis* cells L^-1^ (Fig 2). As cell concentration increases so does the number of cells in the FOV.

### Field validation

**Fig 3:**
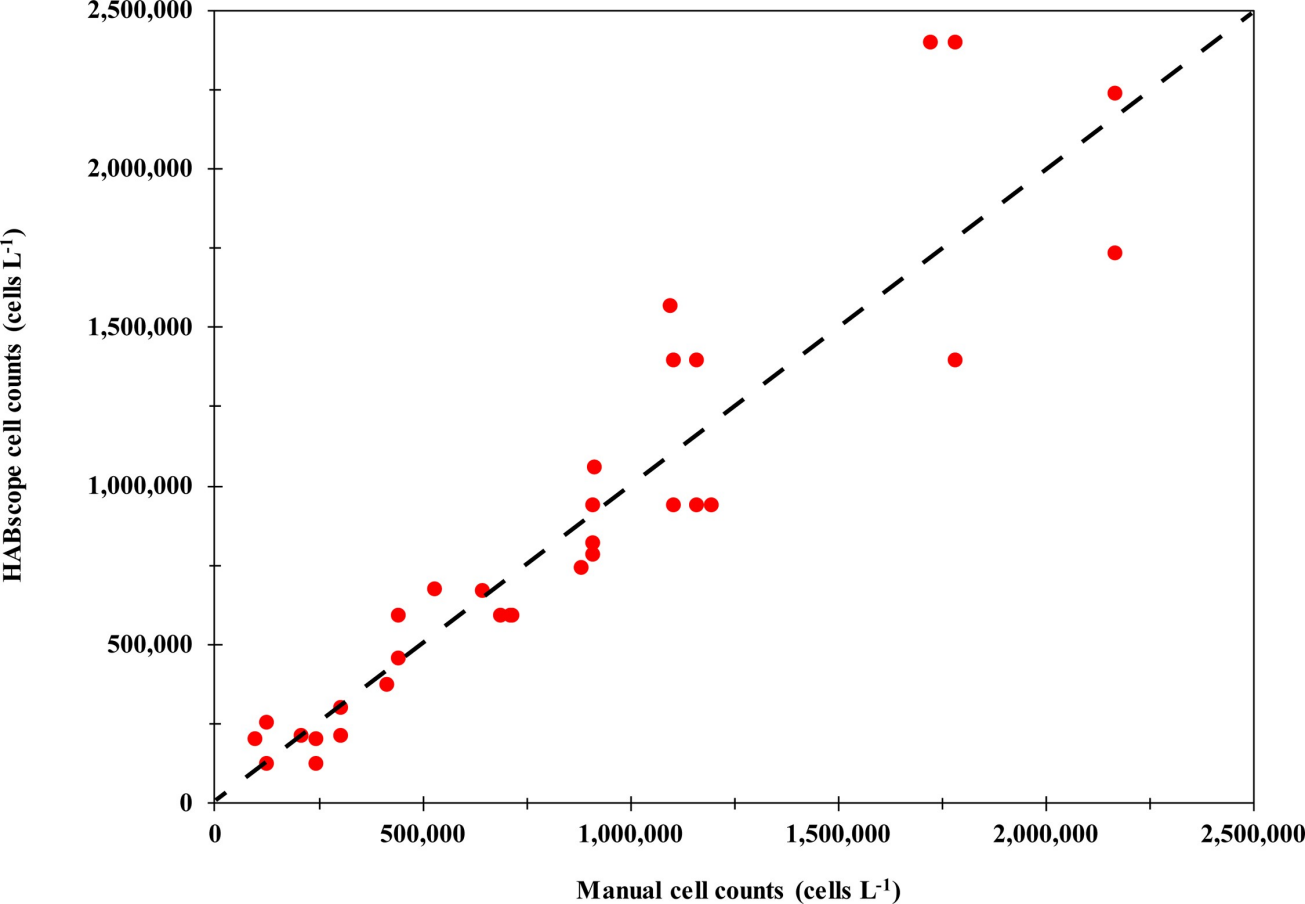
Field validation of HABscope cell counts. A plot showing the HABscope cell counts versus manually counted fixed cells from field-collected samples. The dashed line shows the 1:1 line.

Analysis of HABscope *K*. *brevis* cell abundance data from field samples vs. cell concentrations determined by a laboratory scientists skilled in manual cell counting showed less variation than the HABscope vs. Coulter Counter data using laboratory cultures (Fig 3). The HABscope performed better in the field test than in the laboratory test. There was a negligible positive bias of 3%, and the total error (MAPE) was only 22% over the range from 100,000 to 2,200,000 cells L^-1^. The data were distributed uniformly around the 1:1 line across the range of cell concentrations, although there was a relatively high bias for two of the low cell counts. This bias does include both the error in the manual cell counts and HABscope cell counts. Consider also that manual cell counts have a typical uncertainty of about 10–20% [27].

**Fig 4:**
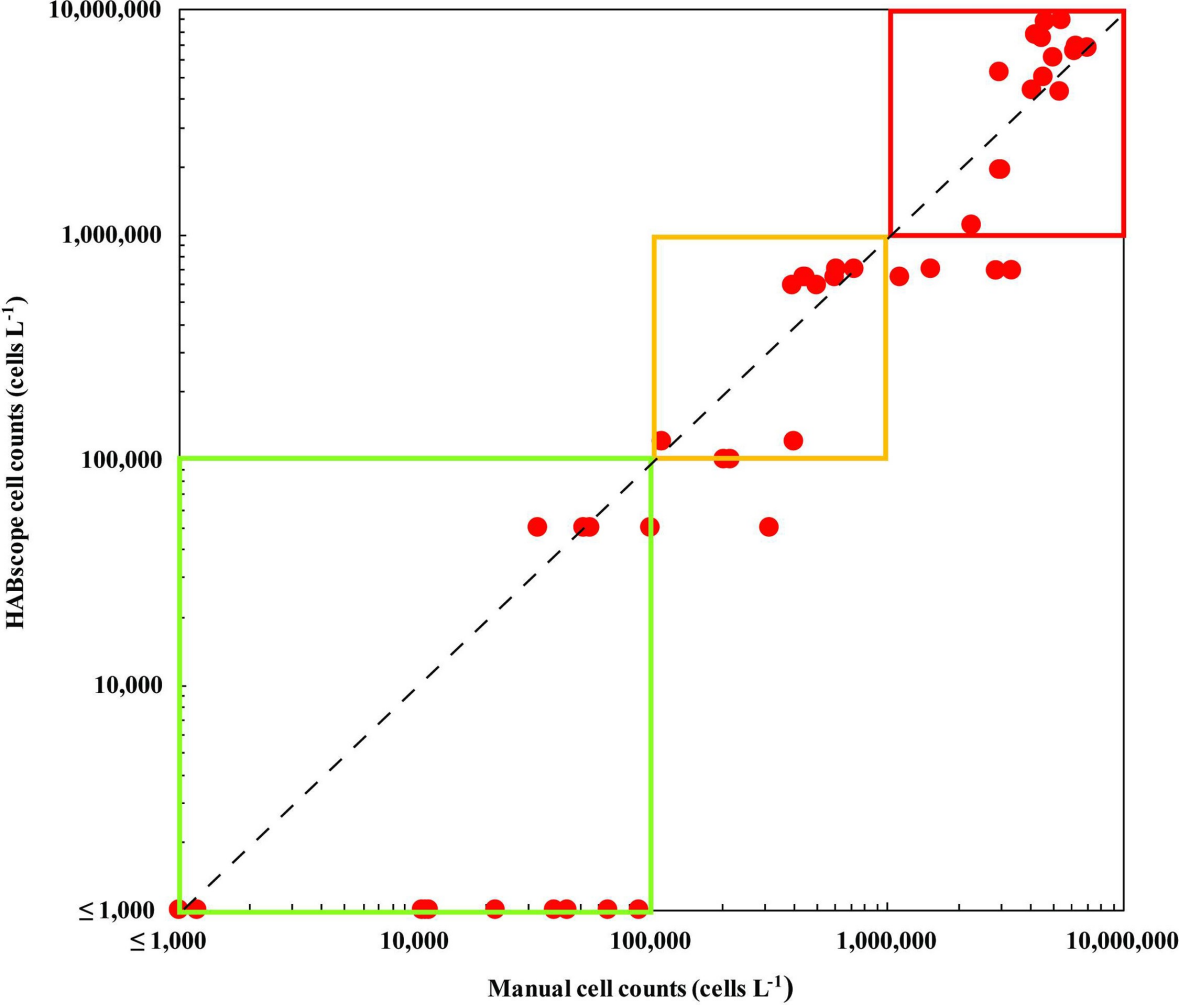
HABscope field application data. The results of the correlation between HABscope estimates of field samples taken by citizen scientist volunteers and cell counts of corresponding samples made by the authors are shown. Zero cell counts were set to 1000 so they could be plotted on a log scale. The green box at lower left shows concentration range for low respiratory risk range, the yellow box in the middle indicates medium-risk, and the red box (upper right) indicates concentrations associated with high-risk. The dashed line is the 1:1 relationship.

The field samples collected by trained citizen scientists had a greater error than the initial deployment by the authors. Excluding the results where no cells were reported by either manual counts or HABscope, the MAPE = 52%, primarily driven by a negative bias of 22% (Fig 4). For a measure of respiratory risk, it is critical that the HABscope system correctly identify *K*. *brevis* abundances with low, medium, or high-risk. The classification matrix showed limited confusion between categories with an overall accuracy of 91% (Table 2). Significantly, there was no confusion between high and low cell abundances. High concentrations were underestimated at a medium level 21% of the time. Of the 46 samples falling into low-risk range based on cell counts, all fell in the same low-risk category based on HABscope estimate. One of the 12 samples identified as falling in the medium-risk category by manual counts was assigned a low-risk estimate by HABscope, while two others were assigned medium-risk at the minimum category limit of 100,000 cells L^-1^. Four of the 19 samples falling into the high-risk category were assigned a medium-risk estimate by the HABscope. The remaining 15 samples fell in the high-risk category as expected. The overall classification accuracy was 94%, with Kappa = 0.89, indicating extremely good agreement.

**Table 2:** HABscope classification matrix. Accuracy comparison of the concentration classes for low, medium, and high respiratory risk.

Microscope Counts	HABscope Low-risk	HABscope Medium-risk	HABscope High-risk	Producer accuracy
Low	46	0	0	100%
Medium	1	11	0	91%
High	0	4	15	79%
User accuracy (reliability)	98%	73%	100%	94%

**Fig 5:**
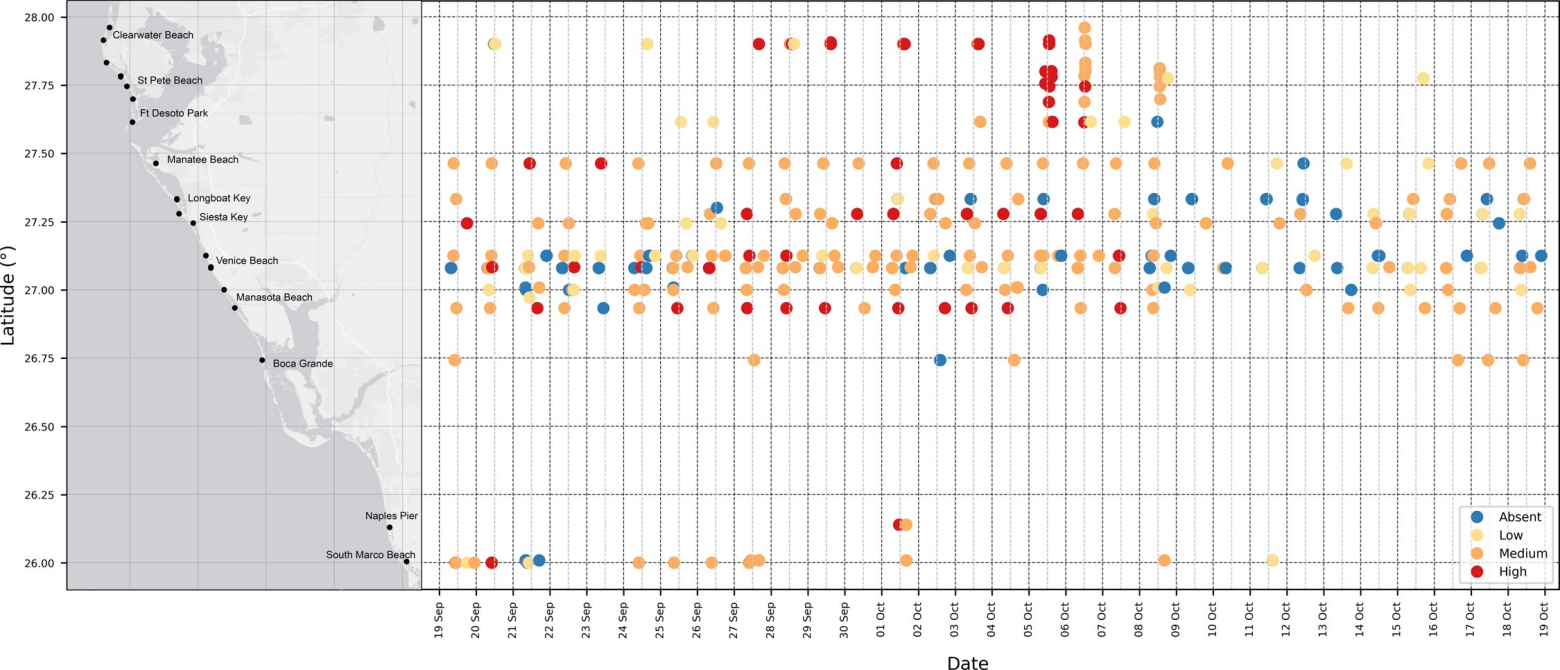
HABscope volunteer monitoring overview. Results of HABscope volunteer monitoring on the west coast of Florida, USA over a 30 day period from 9-19-2018 to 10-19-2018. Each point corresponds to a single sample collected and color denotes *Karenia brevis* cell abundance as absent, low, medium, or high as shown in the figure legend. Y-axis is latitude with locations of the sampling sites marked and X-axis is date.

The ease of use of the HABscope, and dedication of the citizen scientists involved in this study were demonstrated over many weeks (Fig 5). Some beaches were sampled nearly daily, although an interval of 4–6 days was more typical. At one beach in the study (Venice Beach), there were two volunteers, and they frequently coordinated their sampling to collect both morning and afternoon samples. This sampling coverage greatly exceeded sampling frequencies for most of the state and county programs, which collected one sample per week.

## Discussion

Laboratory studies using pure cultures, and an initial field trial by scientists, confirmed that the HABscope system accurately estimates *K*. *brevis* abundance at concentrations ranging from 50,000 to 10,000,000 cells L^-1^ (Figs 2 and 3). The volunteer-generated HABscope cell estimates were more variable, but in general agreed well with the manual counts of *K*. *brevis* (Fig 4). Most importantly, the volunteer samples consistently retrieved the correct category of cell concentration (Fig 5). The primary concern with the volunteer-generated abundances was a tendency to underestimate the actual number of cells. There are several factors that may account for this and need to be addressed to produce better results. One major factor was inconsistency in configuration of the microscope optics. Examples include setting the correct field of view on the iPOD, setting the best focus for the cells, and maintaining the microscope condenser setting for optimal lighting. These skills improved with experience. Each of these factors could contribute to errors in cell abundance estimation. We have not yet been able to field test the system with the Texas Red Tide Rangers due to lack of *K*. *brevis* blooms in their area during the study period. The Rangers have had extensive microscope and HABscope training, so they may demonstrate better initial results.

Given the tendency of the HABscope to sometimes underestimate cell counts, the “gray area” around 100,000 and 1,000,000 cells L^-1^ represents a potential to misclassify counts as medium- or high-risk categories. One simple way to avoid this potential misclassification is to be conservative and have the reviewer of the video select the higher risk category if it is close to the next level in order to err on the side of safety. Another approach is to have the citizen scientists take more videos of the sample to decrease the uncertainty associated with the cell estimate.

A different consideration that may generate errors is the extent of cell motility within a cell population. For instance, it was observed that a portion of the *K*. *brevis* cells in some samples did not swim, potentially limiting recognition by the software. This was most common in samples having more dense *K*. *brevis* concentrations during late bloom stages (which is when most sampling occurred). This could lead to underestimation and will need to be investigated more closely to determine if density or status of a bloom is a factor. As a result, overestimation was infrequent. Other factors expected to interfere with the software’s ability to count *K*. *brevis* cells in natural samples were not an issue. The overall results show little evidence of confusion of *Karenia* with detritus. Similarly, other swimming cells were present in videos, but only rarely counted by the system. The latter issue, however, requires more monitoring in the event that unusual blooms of other algae appear in the area during a *K*. *brevis* bloom.

While this system is sufficient for identifying and mitigating respiratory risk, it does not provide the level of sensitivity required for decisions regarding closure of shellfish beds. Currently, the State of Florida uses an established criterion of 5,000 *K*. *brevis* cells L^-1^ for closure of shellfish beds due to neurotoxic shellfish poisoning (NSP) risks [28]. This threshold concentration is below the detection limit of the HABscope.

This study also demonstrated that a network of citizen scientists could be recruited and deployed in the field during a bloom to take high frequency samples for estimating *K*. *brevis* cell concentrations (Fig 5). There are many capable people willing to take on this role and some can eventually be trained to assist other volunteers and to review a subset of the videos to ensure they meet quality control standards and help expand the system as needed to provide higher resolution coverage of different beaches. The ultimate goal is to provide a respiratory forecast on “Every Beach, Every Day” during ongoing *K*. *brevis* blooms.

The HABscope system is also flexible, and could be trained to recognize other HAB species with unique size or swimming characteristics. One logical candidate is *Pyrodinium bahamense*, a toxic dinoflagellate of particular concern in Florida estuaries. *P*. *bahamense* has an unusual flattened top shape and produces potent neurotoxins known as saxitoxins (STXs) [29]. Blooms of this species are associated with saxitoxin pufferfish poisoning (SPFP), a public risk via tainted fish caught recreationally [30]. They can also cause paralytic shellfish poisoning (PSP) and closure of the shellfish beds, particularly in tropical and subtropical estuarine systems.

The next step in the evolution of the HABscope *K*. *brevis* monitoring system, which is currently underway, is to automatically integrate cell count data from the citizen scientists with high resolution wind forecasts. A prototype web-based display that allows access to the risk predictions for individual beaches via tablet, desktop or cell phone is currently being tested. This system will be operated by the Gulf of Mexico Coastal Ocean Observing System (GCOOS, https://www.gcoos.org), which integrates environmental monitoring data from the coastal states of Texas, Louisiana, Mississippi, Alabama and Florida. GCOOS has the capacity to take input from citizen scientist networks. As envisioned, the HABscope system can be readily integrated into other existing *Karenia brevis* monitoring programs throughout the Gulf of Mexico. For example, members of the Red Tide Rangers, a citizen volunteer bloom monitoring program administered by the University of Texas (https://www.utrgv.edu/csl/public-service/red-tide/index.htm), have already been trained on how to use the HABscope for monitoring *K*. *brevis* the next time a toxic bloom impacts areas along the Texas, USA coast.

In summary, the development of the HABscope provided an accurate, low cost way for volunteers to enumerate cells during an established *K*. *brevis* bloom both temporally and spatially. Conventional monitoring efforts in Florida currently take up to one week for manually counted cell abundance data to be collected and notices provided to the public. During this time, bloom conditions frequently change at the beach, making the delayed report inaccurate. The use of the HABscope by citizen scientist volunteers addresses both the need for near real time bloom data as well as the patchy nature of blooms, eliminating the need for advisories over broad sections of the coast. Instead, local forecasts can be generated specific to individual beaches. Citizen scientists were recruited strategically, to provide the greatest possible coverage along Florida beaches. The accuracy of the data they generated and their demonstrated perseverance in sampling during the bloom demonstrates the power of citizen science to address a major environmental threat to local communities in Florida, and for coastal regions throughout the Gulf of Mexico.

## Supporting information

S1 Video A video of the neural network program estimating *K*. *brevis* cell concentrations in a field sample. (MP4)

S1 File 3D object file for the iPOD adaptor. (STL)

S2 File 3D object file for the microscope adaptor. (STL)
